# Analysis on the risk of myasthenia gravis related to immune checkpoint inhibitors based on the US FDA Adverse Event Reporting System

**DOI:** 10.1002/cam4.6559

**Published:** 2023-09-19

**Authors:** Qingli Kong, Hui Wang, Xiaolei Ren, Yue Zhuo, Jing Peng

**Affiliations:** ^1^ Phase I Clinical Trial Laboratory Affiliated Hospital of Jining Medical University Jining Shandong China; ^2^ Department of Pharmacy Affiliated Hospital of Jining Medical University Jining Shandong China; ^3^ Medical Big Data Center Affiliated Hospital of Jining Medical University Jining Shandong China

**Keywords:** adverse drug reaction reporting systems, antineoplastic agents, computer‐assisted signal processing, immunotherapy, myasthenia gravis, myasthenic syndrome

## Abstract

**Objective:**

To evaluate the risk of myasthenia gravis (MG) associated with immune checkpoint inhibitors (ICI).

**Methods:**

Adverse event (AE) reports related to MG, myasthenic syndrome, and MG crisis for durvalumab, atezolizumab, pembrolizumab, nivolumab, avelumab, and ipilimumab in the US FDA Adverse Event Reporting System (FAERS) from Q1 2004 to Q3 2022 were collected. The proportional reporting odds ratio (PRR) method was used to evaluate the correlation between the six drugs and the three AEs. Statistical significance was defined as having reports ≥3, PRR ≥ 2, and chi‐square (*χ* ^2^) ≥ 4.

**Results:**

A total of 36, 78, 276, 380, 5, and 53 AE reports were collected for durvalumab, atezolizumab, pembrolizumab, nivolumab, avelumab, and ipilimumab, respectively. For myasthenic syndrome, the PRR values reflecting the correlation with the drugs were 27.83 (*χ* ^2^ = 102.66), 26.20 (*χ* ^2^ = 235.67), 44.17 (*χ* ^2^ = 1313.98), 32.09 (*χ* ^2^ = 1229.54), 21.31 (*χ* ^2^ = 151.15), and 0 for durvalumab, atezolizumab, pembrolizumab, nivolumab, avelumab, and ipilimumab, respectively. For MG, the PRR values reflecting the correlation with the drugs were 24.21 (*χ* ^2^ = 682.04), 18.34 (*χ* ^2^ = 900.27), 39.32 (*χ* ^2^ = 7945.15), 26.93 (*χ* ^2^ = 6636.45), 14.73 (*χ* ^2^ = 566.47), and 15.69 (*χ* ^2^ = 54.77) for durvalumab, atezolizumab, pembrolizumab, nivolumab, avelumab, and ipilimumab, respectively. For MG crisis, there were no data for durvalumab, atezolizumab, avelumab, and ipilimumab; the PRR values reflecting the correlation with the drugs were 16.54 (*χ* ^2^ = 225.23) and 9.20 (*χ* ^2^ = 119.14) for pembrolizumab and nivolumab, respectively. All six drugs were statistically correlated with their corresponding AEs.

**Conclusions:**

ICI may lead to ICIs‐associated MG during therapy. Analysis of FAERS data identified signals for AEs of MG with ICI regimens. Practitioners should consider the factors that may increase the likelihood of MG. The findings support a continued surveillance and risk factor identification.

## 1 INTRODUCTION

In the last decade, immune checkpoint inhibitors (ICI) have emerged as a popular anti‐tumor drug and have become the first or second line of treatment for many types of cancer, such as non‐small cell lung cancer, head and neck squamous cell cancer, gastric or gastroesophageal junction adenocarcinoma, colorectal cancer, and melanoma.^1^, ^2^, ^3^, ^4^, ^5^ ICI works by targeting the programmed cell death 1 receptor (PD‐1), programmed cell death ligand 1 (PD‐L1), and cytotoxic T lymphocyte‐associated protein 4 (CTLA‐4), activating effective T cells, and killing tumor cells.^6^, ^7^ However, while ICI can kill tumor cells, they can also over‐activate immune cells, leading to immune‐related adverse events (irAEs). One such serious irAE is myasthenia gravis (MG), a disease caused by neuromuscular junction transmission dysfunction. The most severe manifestation of MG is the myasthenia crisis, which affects the respiratory muscles and requires respiratory support treatment such as mechanical ventilation and intensive care.^8^ ICI‐related MG is a life‐threatening irAE that appears early in ICI treatment. It has low specificity of symptoms, acute onset, and rapid progress, making early diagnosis and intervention critical. Failure to diagnose and treat ICI‐related MG promptly can result in a high mortality rate. Understanding the risks and characteristics of ICI‐related MG is crucial for early identification and effective treatment.

Analyzing adverse event (AE) database information using signal detection methods can help identify potential risk signals of drugs and provide valuable insights for safe and rational clinical use of drugs. This approach can also help overcome the limitations of clinical trials, which may not be able to evaluate all potential drug‐related AEs due to limited sample size and duration of follow‐up.^9^, ^10^ In the process of drug clinical trials, subjects may face various unknown risks, and researchers must provide sufficient protection for their personal rights. By utilizing FDA Adverse Event Reporting System (FAERS) data mining, ethical issues do not need to be considered. The US FAERS database is a valuable source of information for drug‐related AEs that occur both in the United States and abroad. This database provides a real‐world view of drug safety and can help in identifying potential risks associated with specific drugs. In this study, we analyzed the relevant data from the FAERS database to investigate the risk of MG associated with several ICI drugs, including durvalumab, atezolizumab, pembrolizumab, nivolumab, avelumab, and ipilimumab, which are approved for use in the United States and China. By using signal detection methods to analyze the data, we can identify any potential safety concerns associated with the use of these drugs, which can provide valuable information for clinicians and patients in making informed decisions about drug therapy.

## 2 MATERIALS AND METHODS

### 2.1 Data source

**FIGURE 1:**
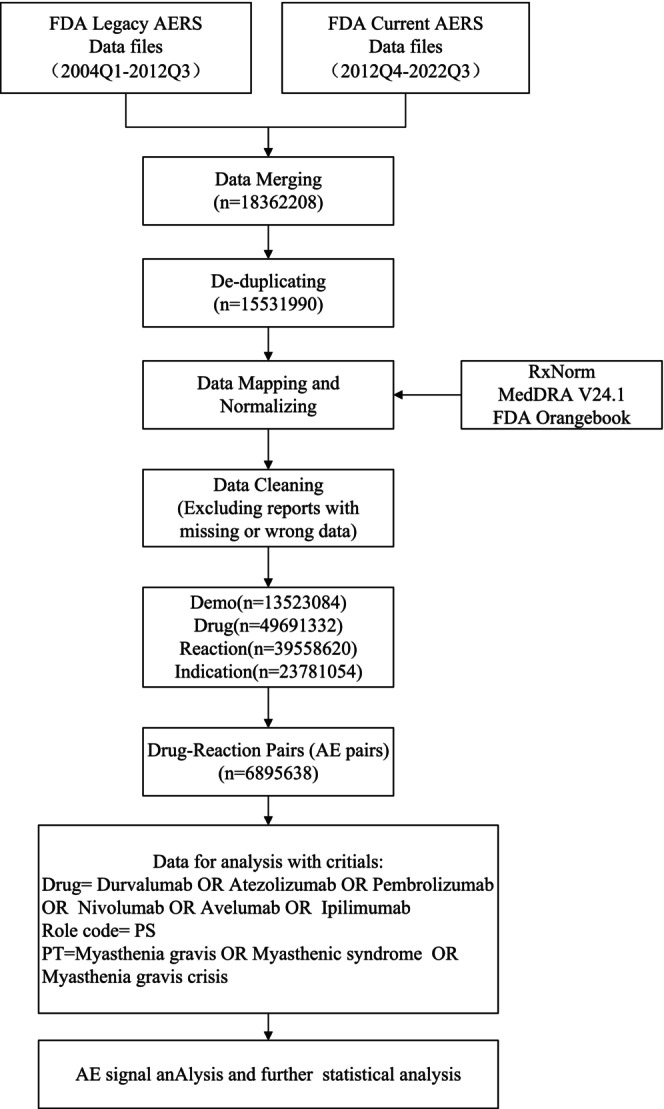
Flow diagram of data extraction and cleaning.

A retrospective pharmacovigilance study was conducted using data from the FAERS database from the first quarter of 2004 to the third quarter of 2022. The data were cleaned and standardized using Python and PostgreSQL database. The study targeted six immunosuppressive agents (durvalumab, atezolizumab, pembrolizumab, nivolumab, avelumab, and ipilimumab) and screened the AE reports related to these drugs.^11^ The AE and medication indications were standardized using the preferred term (PT) in the Medical Dictionary for Regulatory Activities v24.0. The study aimed to extract all AEs related to MG (MG), including its manifestations (enimyastha syndrome) and more severe complications (myasthenia crisis). A self‐designed Excel data extraction table was used to record patient information and AE outcomes. The study recorded patient age, gender, clinical outcomes, reporter's occupation, reporting country, reporting year, and AE occurrence time. The study used R software to statistically compute drug reaction signals. Figure 1 shows the flow diagram of data extraction and cleaning.

### 2.2 Risk signal analysis of ICI‐GM

The study used the proportional reporting ratio (PRR) method to calculate the target AE risk signal based on the imbalance analysis. The PRR and chi‐square (*χ* ^2^) values were calculated using the four‐grid table of the ratio imbalance measurement method. The calculation formula^12^ is as follows: PRR = a/(a + b)/c/(c + d); *χ* ^2^ = ∑(O‐E)2/E, where the number of reports containing both the suspect drug and the suspected adverse drug reaction is called “a”; the number of reported adverse drug reactions associated with the suspect drug (excluding the event of interest) is “b”; a suspect adverse drug reaction with another medication (other than the drug of interest) is called “c”; other adverse drug reactions and medications are included in “d.”

### 2.3 Statistical treatment

The statistical data analysis was conducted using R software (version 4.1.0). Non‐normally distributed data were represented by the median (25% and 75% quantiles) as M (Q1, Q3), while counting data were represented by the frequency (%). The PRR method was used to evaluate the risk of the target drug causing the target AE. A PRR ≥ 2 and *χ* ^2^ ≥ 4 with a number of reports ≥3 indicated a statistical correlation between the target drug and the target AE. A higher PRR indicated a stronger correlation between the drug and AE, thus indicating a stronger risk signal.^13^

## 3 RESULTS

### 3.1 Basic information of ICI‐GM related AE reports

The study used FAERS data to establish a local database of AE reports with six immunosuppressants: durvalumab, atezolizumab, pembrolizumab, nivolumab, avelumab, and ipilimumab as primary suspects. The study focused on AE reports with PTs related to MG, myasthenia syndrome, and myasthenia crisis, and identified a total of 36, 78, 276, 380, 5, and 53 AE reports, respectively. The majority of patients were between 45 and 79 years old, with atezolizumab having the highest percentage of patients over 80 years old (nearly 1/5). More men than women were involved in the reports, and most reporters were doctors. The United States and Japan were the primary reporting countries, and the reporting years were concentrated in the past 5 years with an increasing trend year by year. However, the number of reports of nivolumab, pembrolizumab, and atezolizumab decreased in 2020 but increased in 2022. Avelumab had fewer reported cases. The majority of reported outcomes were hospitalization or prolonged hospitalization, with pembrolizumab having the highest proportion (72.02%). The proportion of deaths ranged from 14% to 40%. See Table 1 for details.

**TABLE 1:** Basic information of reports on myasthenia gravis.

Characteristics *N* (%)	Durvalumab	Atezolizumab	Pembrolizumab	Nivolumab	Avelumab	Ipilimumab
Age group (years)						
<18	0	0	1 (0.46)	1 (0.33)	0	0
19–44	1 (3.13)	0	8 (2.75)	2 (0.67)	0	1 (1.89)
45–64	7 (18.75)	15 (19.15)	54 (19.72)	6 (16.33)	1 (20.00)	14 (26.41)
65–79	20 (56.25)	26 (34.04)	118 (42.66)	193 (50.67)	2 (40.00)	30 (56.60)
>80	1 (3.13)	15 (19.15)	47 (16.97)	51 (13.33)	2 (40.00)	3 (5.60)
Unknown	7 (18.75)	22 (27.66)	48 (17.43)	71 (18.67)	0	5 (9.50)
Gender						
Male	18 (50.00)	43 (55.32)	185 (66.97)	223 (58.67)	2 (40.00)	42 (80.00)
Female	12 (34.38)	20 (25.53)	81 (29.36)	117 (30.67)	3 (60.00)	9 (17.33)
Unknown	6 (15.63)	15 (19.15)	10 (3.67)	40 (10.67)	0	2 (2.67)
Reporter						
Physician	19 (53.13)	58 (74.47)	97 (35.32)	162 (42.67)	4 (80.00)	39 (73.58)
Pharmacist	5 (12.50)	3 (4.26)	15 (5.50)	39 (10.33)	0	4 (7.54)
Other health‐professional	3 (9.38)	0	16 (5.96)	80 (21.00)	0	5 (9.44)
Consumer	1 (3.13)	0	115 (41.74)	34 (9.00)	1 (20.00)	5 (9.44)
Unknown	8 (21.88)	17 (21.28)	33 (11.47)	65 (17.00)	0	0
Outcome						
Death	13 (40.63)	26 (34.04)	84 (30.28)	127 (33.33)	2 (14.28)	61 (18.77)
Disability	1 (3.13)	0	30 (11.01)	23 (6.00)	2 (14.28)	7 (2.15)
Hospitalization—initial or prolonged	19 (59.38)	43 (55.32)	199 (72.02)	262 (69.00)	9 (64.28)	196 (60.31)
Life‐threatening	4 (12.50)	1 (2.13)	61 (22.02)	141 (37.00)	1 (7.14)	60 (18.46)
Other serious (important medical event)	11 (34.38)	41 (53.19)	250 (90.37)	363 (95.66)	0	1 (0.31)
Country						
America	15 (43.75)	22 (27.66)	122 (44.04)	170 (44.67)	0	8 (15.09)
Japan	5 (15.63)	23 (29.79)	61 (22.02)	109 (28.67)	0	34 (64.15)
France	1 (3.13)	3 (4.26)	6 (2.29)	11 (3.00)	0	0
United Kingdom	2 (6.25)	3 (4.26)	9 (3.21)	13 (3.33)	0	2 (3.77)
Australia	2 (6.25)	0	16 (5.96)	6 (1.67)	0	1 (1.88)
Reporting year						
2014	1 (2.78)	0	1 (0.36)	3 (0.79)	0	2 (3.77)
2015	0	0	7 (2.53)	10 (2.63)	0	1 (1.88)
2016	1 (2.78)	2 (2.56)	12 (4.35)	36 (9.47)	0	1 (1.88)
2017	4 (11.11)	3 (3.85)	24 (8.70)	55 (14.47)	0	3 (5.66)
2018	8 (22.22)	8 (10.26)	51 (18.48)	51 (13.42)	1 (20.00)	4 (7.54)
2019	5 (13.89)	9 (11.54)	53 (19.20)	66 (17.37)	1 (20.00)	4 (7.54)
2020	10 (27.78)	12 (15.38)	40 (14.49)	43 (11.32)	0	7 (13.20)
2021	3 (8.33)	12 (15.38)	30 (10.87)	36 (9.47)	1 (20.00)	6 (11.32)
2022	4 (11.11)	31 (39.74)	58 (21.01)	80 (21.05)	2 (40.00)	22 (41.51)

*Note*: *N*, number of reports containing the suspect drug and the suspect adverse drug reaction. %, fraction of the adverse event.

### 3.2 ICI‐GM risk signal analysis

**FIGURE 2:**
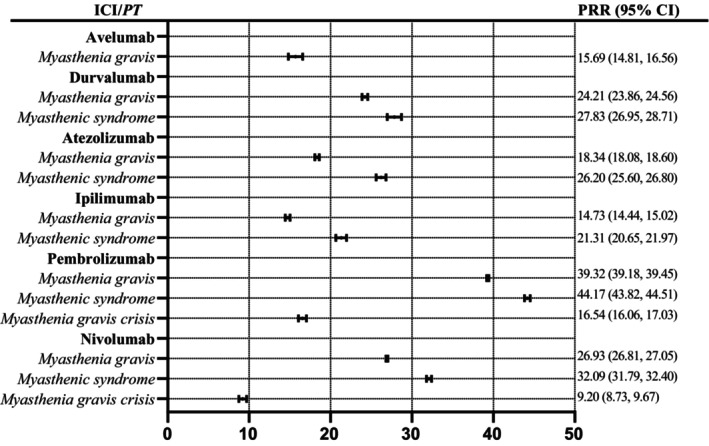
PRR for ICI with myasthenia gravis. ICI, immune checkpoint inhibitors; PRR, proportional reporting ratio. 95% CI, 95% confidence interval.

The study analyzed the risk signals associated with myasthenia syndrome, MG, and myasthenia crisis for six immunosuppressants—durvalumab, atezolizumab, pembrolizumab, nivolumab, ipilimumab, and avelumab. The number of reported cases for each immunosuppressant related to myasthenia varied. The PRR related to myasthenia syndrome was highest for pembrolizumab (44.17), followed by nivolumab (32.09), durvalumab (27.83), atezolizumab (26.20), ipilimumab (21.31), and avelumab (0). The PRR associated with MG was highest for pembrolizumab (39.32), followed by nivolumab (26.93), durvalumab (24.21), atezolizumab (18.34), ipilimumab (14.73), and avelumab (15.69). There were no data available for MG crisis for durvalumab, atezolizumab, avelumab, and ipilimumab. The PRR for MG crisis were highest for pembrolizumab (16.54) and nivolumab (9.20). Based on the analysis, the risk signal intensity causing myasthenia syndrome was highest for pembrolizumab, followed by nivolumab, durvalumab, atezolizumab, ipilimumab, and avelumab. The risk signal intensity causing MG was highest for pembrolizumab, followed by nivolumab, durvalumab, atezolizumab, avelumab, and ipilimumab. The risk signal intensity of myasthenia crisis was highest for pembrolizumab, followed by nivolumab. Please refer to Figure 2 for more details.

### 3.3 Onset times of MG

**FIGURE 3:**
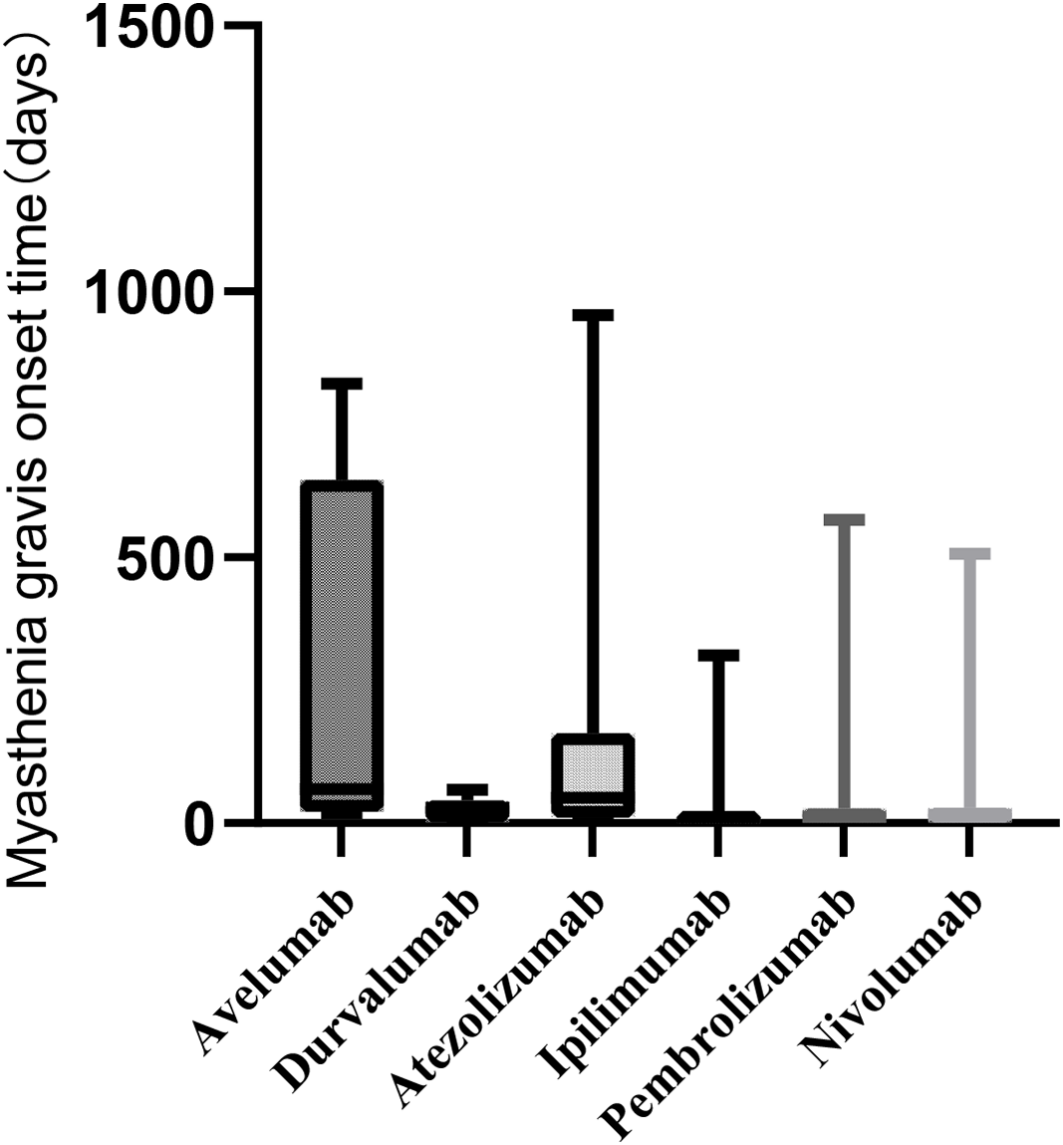
The onset time of myasthenia gravis in patients receiving immune checkpoint inhibitors.

The time from the initiation of durvalumab, atezolizumab, pembrolizumab, nivolumab, avelumab, and ipilimumab treatment to the onset of MG ranged from 1 to 63, 1 to 955, 1 to 571, 1 to 507, 19 to 827, and 1 to 315 days, respectively. The median (Q1, Q3) onset times for MG were 29 (2, 43), 48 (11, 169), 12 (1, 28), 16 (1, 29), 64 (21, 645), and 1 (1, 20) days for durvalumab, atezolizumab, pembrolizumab, nivolumab, avelumab, and ipilimumab, respectively. Please refer to Figure 3 for details.

## 4 DISCUSSION

This study examined the risk of myasthenia crisis and myasthenia syndrome associated with six immunosuppressive drugs at the immune checkpoint. The findings indicated that there were no data on myasthenia crisis in four of the drugs (durvalumab, atezolizumab, avelumab, and ipilimumab). However, the risk signal intensity for myasthenia crisis was highest for pembrolizumab and nivolumab. For myasthenia syndrome, the risk signal intensity was highest for pembrolizumab, followed by nivolumab, durvalumab, atezolizumab, ipilimumab, and avelumab. The study suggests that all six immunosuppressive drugs at the immune checkpoint can lead to MG, and that clinicians should pay particular attention to the use of pembrolizumab due to its strong risk signal strength. These findings emphasize the importance of carefully monitoring patients who receive these drugs for any signs or symptoms of MG, and considering alternative treatment options when appropriate. It is important to note that the study does not necessarily rule out the use of immunosuppressive drugs in combination at the immune checkpoint. However, Antonia et al.^14^ have pointed out that patients using CTLA‐4 inhibitor and PD‐1/PD‐L1 inhibitor together are more likely to have treatment‐related AEs and higher grades of adverse reactions, and the incidence and severity of MG may also be higher. Therefore, clinicians should be vigilant and closely monitor patients for any adverse effects when using these drugs in combination. The study also found that most of the patients who used the six immunosuppressive drugs were between the ages of 45 and 79, and nearly 1/5 of patients using atezolizumab were over 80 years old. Furthermore, approximately 15% of patients using pembrolizumab and nivolumab were over 80 years old, and more men than women were involved in the report. This may be related to the fact that melanoma and NSCLC are more common in the elderly,^15^, ^16^ and highlights the need for clinicians to pay attention to the monitoring of elderly patients when using immunosuppressive agents at the immune checkpoint. Moreover, according to Hasegawa et al.^15^ nivolumab or pembrolizumab may promote disease recurrence in patients with a history of MG. Therefore, it is important for clinicians to carefully evaluate the risks and benefits of using immunosuppressive drugs in patients with a history of MG, and to closely monitor such patients for any signs of recurrence.

In a retrospective study by Gutzmer et al.^17^ it was found that 42% (8/19) of melanoma patients with a history of autoimmune diseases who used PD‐1 inhibitors had a relapse of the original autoimmune diseases. This suggests that a history of autoimmune diseases may be a risk factor for subsequent immune diseases, and clinicians should be cautious when using ICI in the treatment of patients with pre‐existing autoimmune diseases. On the contrary, in a study on the treatment of melanoma patients with CTLA‐4 inhibitors,^18^ it was found that patients with effective tumor remission were more likely to develop autoimmune toxicity. Therefore, oncologists should be highly vigilant for the occurrence of MG in patients who experience tumor remission after ICI treatment. Taken together, these findings highlight the need for close monitoring and careful evaluation of the risks and benefits of using immunosuppressive agents at the immune checkpoint, especially in patients with pre‐existing autoimmune diseases and those who experience tumor remission after ICI treatment.

This study reveals poor outcomes for patients with MG who received ICI. The reported outcomes included hospitalization or prolonged hospitalization, which accounted for more than 55% of patients who took durvalumab, atezolizumab, pembrolizumab, nivolumab, avelumab, and ipilimumab. Furthermore, the number of fatal cases was high, ranging from 14% to 40% for each drug. In contrast to the 2%–10% of patients with spontaneous MG, 50% of those who received ICI had grade 4/5 MG.^19^, ^20^ MG is a serious immune disease of the nervous system that commonly presents with ocular symptoms such as ptosis and/or diplopia, as well as muscle weakness of limbs, dyspnea, and dysphagia. About two‐thirds of patients have severe myodynamia classified as Grade 5 by the Myasthenia Graves Foundation of America, and approximately 45% have rapid progression of life‐threatening respiratory muscle hypodynamia.

This study found that the onset of MG occurred within a range of 1–63 days for durvalumab, 1–955 days for atezolizumab, 1–571 days for pembrolizumab, 1–507 days for nivolumab, 19–827 days for avelumab, and 1–315 days for ipilimumab. The median (Q1, Q3) onset times for MG were 29 (2, 43) days for durvalumab, 48 (11, 169) days for atezolizumab, 12 (1, 28) days for pembrolizumab, 16 (1, 29) days for nivolumab, 64 (21, 645) days for avelumab, and 1 (1, 20) day for ipilimumab. Compared to other irAEs of the immune system, MG often occurs earlier in the course of ICI treatment. Therefore, when using immunosuppressants at clinical checkpoints, it is crucial to be alert and pay close attention to the onset of MG‐related symptoms to avoid rapid disease progression. Another study by Safa et al.^21^ found that most patients who developed MG symptoms after ICI treatment did so after 2 cycles of ICI treatment, with a median time of 4 weeks (ranging from 6 days to 16 weeks), which is consistent with the results of this study. These findings highlight the life‐threatening nature of ICI‐induced MG, which is characterized by nonspecific symptoms, acute onset, and rapid progression.

The clinical manifestations of ICI‐related MG are often nonspecific, making early diagnosis challenging. Patients may experience fatigue, mild muscle pain, and weakness in the limbs, which can be difficult to distinguish from symptoms of advanced tumors and are sometimes overlooked by patients and oncologists. Moreover, patients may not report mild symptoms to clinicians, and may only seek medical attention when the symptoms become obvious, leading to a missed opportunity for timely diagnosis and treatment. To address this issue, clinicians need to carefully monitor the occurrence of MG‐related symptoms during the first 6 weeks of ICI treatment, particularly within 3–4 months of treatment initiation.^22^ According to previous studies, MG tends to develop early during ICI treatment, and may progress rapidly, with a median progression time of 7 days (ranging from 24 h to 60 days) in ICI‐treated patient.^23^ Furthermore, patients with elevated levels of creatine kinase (CK) and/or troponin may have a more rapid disease progression and a higher mortality rate compared to those without such elevations. Myositis and myocarditis are common comorbidities in ICI‐MG, with reported frequencies of 30% and 25%, respectively. Notably, most patients with ICI‐MG die within 6 weeks of the onset of MG symptoms, and those with myositis and/or myocarditis may have a worse prognosis.^19^ Therefore, early detection and prompt management of ICI‐related MG are crucial for improving patient outcomes. According to a study, only 0.9% of patients with iMG have concomitant myocarditis,^24^ whereas iMG is more commonly associated with thymoma. In a study by Hasegawa et al.^15^ it was observed that compared to classical MG, MG caused by ICI has distinct clinical features, such as an early onset during ICI treatment and a variable disease severity. Furthermore, the frequency of MG crisis increases and CK levels rise significantly when the condition worsens. Thus, it is important for clinicians to closely monitor patients for muscle symptoms and CK values before and after administering immunosuppressive drugs at the checkpoint. Although the diagnosis of ICI‐MG can only be confirmed after considering other relevant differential diagnoses, the close temporal relationship between myasthenia symptoms and the administration of ICI can aid clinicians in making a prompt diagnosis and initiating appropriate treatment.

MG is a medical condition that can cause a serious complication called myasthenia crisis, which affects the respiratory muscles and can lead to a high mortality rate.^25^ Treatment for myasthenia crisis typically involves respiratory support, such as mechanical ventilation and intensive care.^26^ Studies have found that irAEs related to ICIs such as cytotoxic T‐lymphocyte associated protein 4 (CTLA‐4) inhibitors and PD‐1 inhibitors can also affect the nervous system. The incidence of nervous system irAEs related to CTLA‐4 inhibitors was found to be 6.1%, while the incidence of nervous system irAEs related to PD‐1/PD‐L1 inhibitors was 3.8%. When both CTLA‐4 and PD‐1 inhibitors were combined, the incidence of nervous system irAEs increased to 12%.^27^ However, it is important to note that the true incidence rate of nervous system irAEs may be underestimated, as they could be overlooked, misdiagnosed, or underreported. As more and more patients with early tumors receive ICI treatment, the number of rare or severe irAEs may increase, which will require oncologists to be vigilant in their monitoring and management of these potential side effects.

Early identification and treatment of clinical manifestations related to MG are crucial in reducing the occurrence of myasthenia crisis, mortality, disability rate, and prolonged hospitalization. The incidence of ICI‐related MG may continue to rise due to the increasing use of ICIs for cancer treatment. Therefore, early recognition of MG is a critical step toward developing evidence‐based treatment algorithms that can improve clinical outcomes.^28^ Further research in this area is needed not only to inform treatment decisions but also to mitigate the risks associated with the use of ICI treatment. By understanding the mechanisms underlying ICI‐related MG, healthcare providers can better identify and manage this potentially life‐threatening condition. In addition, ongoing research can help to identify risk factors for the development of ICI‐related MG and guide the selection of patients who may be at higher risk for this AE. Overall, continued research efforts are essential for improving the safety and efficacy of ICI therapy in cancer patients.

Hochberg et al.^29^ used data from the FAERS database to analyze the risk signals of drug‐induced AEs. The authors recommended using the FAERS database for signal mining of drug‐induced AE risks, but emphasized that strict standards must be followed to ensure the accuracy of the results. The study identified five criteria that need to be met when analyzing data from the FAERS database. Firstly, data for the drug‐event combination being studied must be free of extreme duplication. Follow‐up data in the FAERS database can lead to the reporting of AEs caused by the same drug in the same patient by different persons at different times, resulting in duplicate data and expanding risk signals. Secondly, the patient population needs to have the same age and gender. Thirdly, the drugs must be used for a similar spectrum of indications. Fourthly, the drugs must be used along with a similar spectrum of concomitant medications. Lastly, drugs that have been on the market for more than 2–3 years should not be compared with drugs in the peri‐approval period. In the case of the six drugs analyzed in this study, they met the five criteria mentioned above, indicating that the strength of the risk signal for MG may represent the incidence of drug‐related MG. Overall, the study underscores the importance of adhering to strict standards when using the FAERS database for signal mining of drug‐induced AE risks.

The FDA's Adverse Event Reporting System (FAERS)^30^ is a database that contains AE reports, medication errors, and product quality complaints submitted by healthcare professionals, manufacturers, consumers, and patients to the Food and Drug Administration. There is information about demographics, drugs, and reactions in every report. The primary suspected drug is identified in each report, and other drugs taken by the patient may also be listed.

Hochberg et al.^29^ analyzed the FAERS database to calculate the risk signals of drug‐induced AEs. They found that these risk signals were consistent with the incidence of drug‐induced AEs and encouraged the use of the FAERS database for analysis of drug‐induced AE risks. Similarly, Peng et al.^31^ developed a study based on the FAERS database, using signal detection methods to mine AE database information. The study aimed to assist in discovering drug safety signals, providing reference for drug risk management and evaluation, and compensating for the limitations of evaluation methods in drug clinical trials.

Overall, researchers continue to monitor and identify risk factors for AEs using the FAERS database. Its use can assist in discovering drug safety signals and help in the management and evaluation of drug risks. However, it is important to adhere to strict standards when analyzing data from the FAERS database to ensure the accuracy of the results. While the FAERS database is a valuable resource for identifying AEs related to drug use, it also has some limitations. One of the main limitations is underreporting, missed reports, and incomplete reports. Additionally, the signals mined from the database may be underestimated due to various factors. Therefore, more research is needed to confirm the conclusions drawn from data analysis in the FAERS database. However, despite these limitations, the FAERS database remains a valuable tool for accessing real‐world data. It can be used to detect and evaluate adverse drug reactions, provide guidance for rational and safe drug use for patients, and ultimately help to reduce the occurrence of related complications. In conclusion, while the FAERS database has its shortcomings, it is still a valuable resource for researchers and healthcare professionals. The database can provide important insights into drug safety and can contribute to improving patient outcomes.

## AUTHOR CONTRIBUTIONS

**Qingli Kong:** Conceptualization (lead); data curation (lead); investigation (lead); project administration (lead); resources (lead); writing – original draft (lead); writing – review and editing (lead). **Hui Wang:** Writing – review and editing (equal). **Ren Xiaolei:** Conceptualization (lead); data curation (lead); project administration (equal). **Zhuo Yue:** Investigation (equal); methodology (equal); validation (equal). **Peng Jing:** Formal analysis (equal); investigation (equal); methodology (equal); resources (equal); software (equal); supervision (equal).

## FUNDING INFORMATION

This research was partially supported by the Young Research Foundation of Shandong Pharmaceutical Association and Tianji Health Care (hlyy 2021‐03) and by the Key research and development program of Jining (2022YXNS067).

## CONFLICT OF INTEREST STATEMENT

It is hereby declared by the authors that the research was conducted without any commercial or financial relationships that might be construed as conflicts of interest.

## Data Availability

Detailed information about the original contributions of the study can be found in the article materials, or the corresponding authors may be contacted if further inquiries are necessary.
